# The critical role of physical frailty and function on depressive symptoms among community-dwelling older adults in China: A cross-sectional study

**DOI:** 10.3389/fpsyt.2023.1134427

**Published:** 2023-02-15

**Authors:** Qun Wang, Dan Song, Qiuru Lin, An Tao, Yao Zhang, Xilin Li, Xichenhui Qiu

**Affiliations:** ^1^School of Nursing, Shenzhen University, Shenzhen, China; ^2^Department of Nursing, Shenzhen Qianhai Shekou Free Trade Zone Hospital, Shenzhen, China; ^3^The Nethersole School of Nursing, The Chinese University of Hong Kong, Hong Kong, Hong Kong SAR, China

**Keywords:** physical frailty, physical function, older adults, cross-sectional study, depressive symptom

## Abstract

**Introduction:**

With rapid population aging in China, geriatric depression has imposed a heavy burden on public health and society. This study aimed to investigate the prevalence and influencing factors of depressive symptoms among community-dwelling older adults in China. The findings of this study will contribute to the early detection and effective interventions for older adults with depressive symptoms.

**Methods:**

A cross-sectional study was conducted among people aged ≥65 years old in urban communities in Shenzhen, China, in 2021. This study assessed depressive symptoms (Geriatric Depression Scale-5, GDS-5), physical frailty (FRAIL Scale, FS), and physical function (Katz index of independence in the Activities of Daily Living, ADL). Multiple linear regression was used to examine potential predictors of depressive symptoms.

**Results:**

A total of 576 participants aged 71.73 ± 6.41 years were included in the analysis. More than half of them were females (53.0%). The average score of GDS-5 was 0.57 ± 1.11, with 78 (13.61%) participants with depressive symptoms (≥2). The mean scores of FS and ADL were 0.80 ± 1.08 and 9.49 ± 1.67, respectively. The final regression model indicated that those who were living alone, less satisfied with their personal life, frailer, and with worse abilities in ADL had a higher level of depression symptoms (R^2^ = 0.406, *p* < 0.001).

**Conclusion:**

The prevalence of depressive symptoms is high in this urban community-dwelling older adults in China. Given the critical role of frailty and ADL on depressive symptoms, specific attention to psychological support should be paid to older adults who are living alone and with poor physical conditions.

## 1. Introduction

In the last few decades, most countries in the world have experienced increased life expectancy of population and a remarkably increased number of older adults. According to data from World Population Prospects, by 2050, one in six people in the world will be over age 65 (16%), up from one in 11 in 2019 (9%). By 2050, one in four persons could be aged 65 or over (1). About 15% of older adults suffer from mental disorders, among which depression is the most prevalent. As reported by the WHO (2), seven percent of older adults have depressive symptoms, which is the second most widespread burdensome health issue following ischemic heart disease (3). Depressive symptoms among older adults have become a global public health issue. Thus, identifying depressive symptoms and its related risk factors is important.

Previous studies have explored environmental, social, and biological risk factors for depressive symptoms (4). One of these risk factors is frailty. Frailty is regarded as the increased vulnerability of developing dependency and/or death when people, especially the older people, are exposed to stressors (5). Previous studies have suggested that depressive symptoms may be associated with frailty. Soysal et al. (6) conducted a meta-analysis of 14 studies (10 cohort studies and four cross-sectional studies) and found that older adults with depressive symptoms are more likely to become frail compared with those without depressive symptoms (odds ratio [OR] = 2.99, 95% confidence interval [CI] = 2.19–4.08). Several other studies have shown a bidirectional relationship between depressive symptoms and frailty (7–9).

Physical function usually refers to voluntary movements conducted through muscular contractions that allow individuals to interact with the environment (10). It includes various behaviors, like simple motor tasks requiring small muscle groups and complex actions involving interactions between muscle groups and physiological systems (10). Physical function can be measured through determining one's ability to do tasks linked to independent living or quality of life (11), such as the measurement of activities of daily living (ADL) (12). Impaired in physical function is associated with adverse health outcomes, such as mortality, disability, hospitalization, falls, and frailty (13–18). Previous study suggested that the physical function decline may be a risk factor of depression (19). However, little attention was paid to the relationship between physical function and depressive symptoms among older adults.

Previous studies focused on the single role of either frailty or physical function on depressive symptoms among older adults. Few studies explored the roles of both physical function and ADL on depressive symptoms among older adults. Thus, this study sought to investigate the prevalence of depressive symptoms and explore the influences of both frailty and physical function on depressive symptoms among community-dwelling older adults in China.

## 2. Methods

### 2.1. Study design and sample

The study adopted a cross-sectional design. Participants were recruited using convenience sampling method. Older adults from five urban communities in Shenzhen, China were invited to participate in this study through posters and community chat groups between November 2020 and July 2021. The inclusion criteria were people who: (1) age above 65 years old and (2) were able to offer informed consent. The exclusion criteria were people who had: (1) clinical diagnosis of mental diseases, such as bipolar disorder and schizophrenia or (2) serious visual, speech, or hearing impairments.

The required study sample size for this study was estimated based on the power analysis for multiple regression (20). A minimum of 442 participants was needed to achieve a small effect size (*r* = 0.2) with 90% at a power at 5% significance level (20). We used the “Multiple Regression using Effect Size” in IBM SPSS version 25.0 (IBM Corp., Armonk, NY) to calculate the sample size. Taking into account a 20% attrition rate, at least 553 participants were needed for this study.

### 2.2. Data collection

The study was approved by the Ethics Committee of the Medical College of Shenzhen University, China. Before the start of the study, all information about the research project was explained to the potential participants. The potential participants were informed that they had the right to withdraw at their will and at any time and that their participation was voluntary. Written consent was obtained from each participant before the start of data collection. The survey was performed by trained researchers, and data were collected through face-to-face interviews. All data was kept strictly confidential. The study followed the principles outlined in the Declaration of Helsinki.

### 2.3. Measurement

A self-designed questionnaire was used to collect participants' sociodemographic information, including age, gender, marital status, educational level, living status, working status, monthly income, drinking status, smoking status, and medical history. Satisfaction with personal life and health was measured with a visual analog scale (VAS) with a score range of 0–100, with 0 indicating the least satisfaction and 100 indicating the most satisfaction.

#### 2.3.1. Geriatric depression scale-5

GDS-5 is one of the mostly employed screening tools for measuring depressive symptoms. It was developed by Hoyl et al. (21) based on the 15-item Geriatric Depression Scale (21, 22). The five items are scored as 0 or 1, and the total score ranges from 0 to 5. A higher score indicates a greater level of depressive symptoms. A GDS-5 score of ≥2 indicates the existence of depressive symptoms (21). The Chinese version revealed good psychometric properties among older Chinese people (23, 24). To achieve greater statistical power, the current study treated the total score of the GDS-5 as continuous data and the dependent variable in the regression analysis.

#### 2.3.2. The FRAIL scale

The FS is a widely used frail assessment tool for older adults developed from the Frailty Index and Cardiovascular Health Study Index (25). FS has five questions on exhaustion, resistance, walking, diseases, and unintentional weight loss. Each question is answerable by “yes” or “no” with a score of 0 or 1, respectively. The total score ranges from 0 to 5. Previous study suggested the classification for FS score as following: robust (0), pre-frail (1–2), or frail (≥3) (25). The Chinese version of FS revealed a good reliability and validity for frailty assessment in older Chinese adults (26).

#### 2.3.3. Katz index of independence in activities of daily living

In the current study, physical function was measured using Katz ADL, which is a widely used instrument for assessing the daily living ability of older adults (27, 28). Katz ADL includes six dimensions: feeding, dressing, bathing, transfer, going to the toilet, and continence. The scale employs a three-level scoring approach, with a total score ranging from 0 to 12. A higher total score in Katz ADL indicates a better daily activity ability. The Chinese version of Katz ADL has been widely applied in geriatric care and research with satisfactory performance in validity and reliability (29, 30).

### 2.4. Statistical analyses

The categorical variables in this study were presented as percentages and frequencies, and continuous variables were presented as mean ± standard deviation (SD). Moreover, Pearson's correlation, independent t-test, and one-way analysis of variance (ANOVA) were conducted for univariate analyses between depressive symptoms and other potential variables. Variables in the univariate analysis with *p* < 0.1 (31) were included as independent variables in the stepwise multiple linear regression analysis to explore influencing factors of depressive symptoms. All statistical tests were two-sided, and *p* < 0.05 was considered statistically significant. All statistical analysis was conducted using the IBM SPSS version 25.0 (IBM Corp., Armonk, NY).

## 3. Results

### 3.1. Characteristics of participant

The current study invited 649 old adults. Finally, a total of 576 provided written consent and competed the questionnaires, with a response rate of 88.75%. Their sociodemographic characteristics were presented in Table 1. The age of the participants ranged between 65 and 96 years old with an average of 71.73 years old (SD = 6.4). Almost half of the participants were females (305, 53%) and had a middle school education or above (314, 54.5%). Most of the participants were married (85.42%), living with their spouse or children (530, 92.0%), and were retired (456, 79.17%). About 60% of the participants had <3,000 RMB monthly income. Only a small percentage of the participants drank alcohol (27, 4.69%) or smoked (71, 12.39%). The prevalence of hypertension and diabetes in the population was 47.4 and 19.10%, respectively.

**Table 1:** Sociodemographic characteristics of the participants (*N* = 576).

**Characteristics**	**Mean/*n***	**SD/%**
Age (years)^†^ (range 65–96)	71.73	6.409
**Gender**		
Male	271	47.00%
Female	305	53.00%
**Marriage**		
Married	492	85.42%
Bereaved spouse	79	13.72%
Divorced	5	0.86%
**Education**		
Illiterate	68	11.81%
Primary School	194	33.68%
Junior High School	124	21.53%
High School	116	20.14%
College and above	74	12.85%
**Residence status**		
Living alone	36	6.25%
Living with spouse	138	23.96%
Living with children	173	30.03%
Living with spouse and children	219	38.02%
Other	10	1.74%
**Job**		
Retired	456	79.17%
Working	14	2.43%
Other	106	18.40%
**Monthly income**		
< 500 yuan	146	25.35%
(500, 1,000) yuan	82	14.24%
(1,000, 3,000) yuan	117	20.31%
(3,000, 5,000) yuan	123	21.35%
≥5,000 yuan	108	18.75%
**Drinking**		
Never	377	65.45%
Occasionally	140	24.31%
Quit drinking	32	5.56%
Regular drinking	27	4.69%
**Smoking**		
Never	411	71.73%
Quit smoking	91	15.88%
Smoking	71	12.39%
**Hypertension**		
No	303	52.60%
Yes	273	47.40%
**Diabetes**		
No	465	80.73%
Yes	110	19.10%
Satisfaction to one's life (range 2–100)	85.51	14.21
Satisfaction to one's health (range 0–100)	77.05	17.42
**FRAIL scale**		
**Total score** ^†^	0.80	1.08
Frail	50	8.74
Pre-frail	211	36.89
Non-frailty	311	54.37
**Geriatric depression scale**		
Total score^†^ (range 0–5)	0.57	1.11
With depressive symptom (GDS ≥ 2)	78	13.5
Without depressive symptom (GDS < 2)	498	86.5
**Activities of daily living**		
Total score^†^ (range 0–10)	9.49	1.67

Data are presented as N (%) for categorical variables or mean ± standard deviation (SD) for continuous variables.

† indicates continuous variables presented as mean and standard deviation (SD).

The participants' mean GDS-5 score was 0.57 ± 1.11, with 13.61% (*n* = 78) of the participants having depressive symptoms (≥2). The mean FS and Katz ADL scores were 0.80 ± 1.08 and 9.49 ± 1.67, respectively. In addition, 211 participants were prefrail and 50 older adults were frail.

### 3.2. Depressive symptoms among different groups of participants

In the correlation analyses (Table 2), the GDS score was significantly positively associated with FRAIL score (*r* = 0.554, *p* < 0.001) and age (*r* = 0.223, *p* < 0.001) and negatively associated with Katz ADL level (*r* = −0.531, *p* < 0.001), satisfaction with personal health (*r* = −0.392, *p* < 0.001), and satisfaction with personal life (*r* = −0.374, *p* < 0.001). As indicated by independent *t*-test or one-way ANOVA, the levels of depressive symptoms did not show statistical differences among participants with different gender, education, job, or smoking habits. In comparison, participants with different characteristics of marriage (*p* < 0.01), living status (*p* < 0.001), monthly income (*p* < 0.01), hypertension (*p* < 0.01), and diabetes (*p* < 0.05) revealed significant differences in depressive symptoms. Table 3 presented the GDS-5 scores among different groups of participants.

**Table 2:** Pearson's correlations analyses for levels of depressive symptoms.

**Variables**	**GDS-5 total score**	
	***r***	***p***
Age^**^	0.223	< 0.001
Satisfaction with personal life^**^	−0.374	< 0.001
Satisfaction to one's health^**^	−0.392	< 0.001
FS_total score^**^	0.554	< 0.001
Katz ADL_total score^**^	−0.531	< 0.001

^***^ *p* < 0.001.

GDS, Geriatric Depression Scale; FS, FRAIL Scale; ADL, activities of daily living.

**Table 3:** *T*-test or one-way ANOVA of research variables.

**Variables**	***N* (%)**	**Depressive symptoms** ** (Mean ±SD)**	***t* or *F***	***p***
**Gender**			1.011	0.479
Male	271 (47.00)	0.619		
Female	305 (53.00)	0.525		
**Marriage** ^†^ ^***^			9.458	< 0.001
Married	492 (85.42)	0.494		
Bereaved spouse	79 (13.72)	1.064		
Divorce	5 (0.86)	0.200		
**Education** ^†^			0.432	0.786
Illiterate	68 (11.81)	0.500		
Primary School	194 (33.68)	0.505		
Junior High School	124 (21.53)	0.607		
High School	116 (20.14)	0.626		
College and above	74 (12.85)	0.569		
**Living status** ^†***^			5.665	< 0.001
Living alone	36 (6.25)	0.971		
Living with spouse	138 (23.96)	0.674		
Living with children	173 (30.03)	0.705		
Living with spouse and children	219 (38.02)	0.309		
Other	10 (1.74)	1.000		
**Job** ^†^			1.448	0.236
Retired	456 (79.17)	0.605		
Working	14 (2.4)	0.214		
Other	106 (18.40)	0.462		
**Monthly income** ^†**^			3.539	0.007
< 500 RMB	146 (25.35)	0.603		
(500, 1,000) RMB	82 (14.24)	0.185		
(1,000, 3,000) RMB	117 (20.31)	0.547		
(3,000, 5,000) RMB	123 (21.35)	0.623		
≥5,000 RMB	108 (18.75)	0.776		
**Drinking** ^†^			2.224	0.084
Never	377 (65.45)	0.541		
Occasionally	140 (24.31)	0.529		
Quit drinking	32 (5.56)	1.065		
Regular drinking	27 (4.69)	0.592		
**Smoking** ^†^			0.021	0.979
Never	411 (71.73)	0.572		
Quit smoking	91 (15.88)	0.556		
Smoking	71 (12.39)	0.592		
**Hypertension** ^***^			−2.983	< 0.001
No	303 (52.60)	0.439		
Yes	273 (47.40)	0.713		
**Diabetes** ^**^			−2.312	0.004
No	465 (80.73)	0.517		
Yes	110 (19.10)	0.789		

Categorical data of participants' characteristics. SD, standard deviation;

† ANOVA, analysis of variance.

* p < 0.05;

** p < 0.01;

*** p < 0.001.

### 3.3. Predictors of depressive symptoms

The multiple linear regression was conducted to explore the potential predictors of depressive symptoms in older adults. The GDS-5 score was the dependent variable. All variables with *p* < 0.1 in the univariate analysis (age, living status, marital status, alcohol use, monthly income, satisfaction with personal life, satisfaction with personal health, hypertension, diabetes, total Katz ADL score, and total FRAIL score) were included as independent variables in the stepwise multiple linear regression analysis. The linear correlations between continuous independent variables and dependent variables, residual analysis. and multicollinearity was checked carefully. The statistics of 95% CIs, standard error (SE), and beta were computed. All tolerance values were more than 0.735, and all Pearson correlation coefficients between independent variables were < 0.7. Therefore, the multicollinearity could be excluded. The Durbin–Watson value for the multiple linear regression model was 2.006, implying that the residuals had independency. The scatter plots, P–P plots, and histogram reflect that the residuals conformed to the homogeneity of variance and normal distribution.

The final regression model included four predictors: living alone, satisfaction with personal life, FS score, and Katz ADL score, which could explain 40.6% of the variation in depressive symptoms (*R*^2^ = 0.406, *p* < 0.001; Table 4). The final regression model indicated that those who were living alone, less satisfied with their personal life, frailer, and with worse ADL abilities had a higher level of depressive symptoms.

**Table 4:** Results of multiple linear regression analysis for depressive symptom levels.

**Variables**	**Coefficient**	**Standardized coefficient**	**95% CI**		***p***
Living alone	0.305	0.073	0.027	0.584	0.032
Satisfaction to personal life	−0.013	−0.164	−0.018	−0.008	< 0.001
ADL_total score	−0.218	−0.331	−0.268	−0.167	< 0.001
FS total score	0.320	0.324	0.244	0.396	< 0.001

F = 89.70, R^2^ = 0.406, adjusted R^2^ = 0.401, p < 0.001.

FS, FRAIL Scale; ADL, activities of daily living.

## 4. Discussion

This study aimed to investigate the prevalence and the risk factors of depressive symptoms among older Chinese community-dwelling adults. Age, marital status, living status, monthly income, presence of hypertension or diabetes, satisfaction with personal life or health, frailty, and ADL were associated with the prevalence of depressive symptoms. Frailty, ADL dysfunction, old age, and living alone were independent risk factors for the incidence of depressive symptoms among older adults, which accounted for 40.6% of the variation in depressive symptoms.

Our findings indicated the prevalence of depressive symptoms among the studied community-dwelling older adults in Shenzhen was 13.61%. The current depressive symptoms prevalence in Shenzhen was lower compared with the findings in Central China (14.04%) (32) and Western China (19.6%) (33). Our result was more similar to that of a national cohort study in Japan (34). Nevertheless, depressive symptom has developed to be a comparatively common public health issue among older individuals worldwide. The consistency and variances among studies might be attributed to differences in depressive symptoms screening tools, population distributions, and geographic characteristics. The current participants were recruited from urban communities in Shenzhen. Unlike the studies in Central or Western China, Shenzhen is a special economic zone in Southern China with highly developed economic condition. The economic and demographic structure of Shenzhen are more like that in Japan.

This study reflects that the depressive symptoms prevalence among older adults increases with age. The research result is consistent with findings of a previous study (35). One possible explanation might be an age-related chronic illness. Older adults are more likely to experience more serious chronic diseases that increase with age, which might also enhance the risk for depressive symptoms and physical frailty (36). In addition, the emergence of depressive symptoms is associated with the greater prevalence or possibility of physical disorders and chronic illnesses, such as stomach problems, vision problems, heart diseases, and diabetes (37, 38). The prevalence of these chronic diseases might contribute to increased vulnerability (39). Furthermore, the excessive worry of older adults about physical illnesses would have a more profound negative influence on their mental state, which may further trigger depressive symptoms (40). Moreover, chronic pain, especially if combined with poverty, social stress, and social isolation, is more likely to induce depressive symptoms among older adults (41). Compelling evidence has suggested that older adults with severe comorbid symptoms are more likely to become frail than those with moderate/mild comorbid symptoms (42). However, these analyses were conducted in an exploratory manner with a small sample size; thus, a careful interpretation of these findings is warranted.

The main finding of this study indicated that older adults who are living alone, less satisfied with personal life, more physical frail, and with worse ADL abilities have a higher level of depressive symptoms. The result illustrated the critical roles of physical frailty and physical function on depressive symptoms. Many studies have shown that depression interacts with frailty in older adults (8). According to a meta-analysis conducted in the United Kingdom, the overall prevalence of depression is 38.6% among 8,023 individuals who felt frail. Among 2,167 depressed individuals, the prevalence of frailty is 40.40%. Furthermore, the prevalence of depression is four times higher in frail older adults than in non-frail older adults (6). These findings consistently indicated that an increase in frailty may lead to the rise in geriatric depression. According to Woods et al. (43), frailty as a geriatric syndrome is influenced by multiple factors, including depressive symptoms, smoking, obesity, and being underweight. The accompanying weakness, low activity tolerance, and pain may lead to functional dependence or disability, which causes helplessness and sadness and accelerates the onset of depression. In addition, depression may cause frailty due to a depression-induced sedentary lifestyle, weight loss, and increased risk of falls, leading to an increased risk of frailty (44).

Although the exact mechanisms are unclear, some potential underlying mechanisms between depression and frailty may be similar. First, a meta-analysis (6) showed that frailty has a positive correlation with inflammatory parameters, such as interleukin-6 (IL-6) and C-reactive protein (CRP) in cross-sectional studies. IL-6 and CRP also increase in patients with anxiety and depression (45–48). Inflammatory cytokines are related to the decline in muscle strength and mass (49), negatively influencing mental health and brain activity in basic studies (50). Hence, inflammation may be one of the pathogeneses for depression and frailty. However, the exact mechanisms remain to be explored. Chen et al. (51) found that frailty incidence can be reduced by supplementing vitamin D and protein in diets and doing strengthening exercises, such as aerobic exercise and impedance exercise, which is conducive to reduce depression prevalence. Thus, for older community-dwelling adults, the impairment of physical function and frailty are among the risk factors for depression. Depression prevalence in older adults might be reduced by improving the risk factors for frailty.

Furthermore, a regression model indicated that living alone is an underlying predictor for depression, which accorded with previous research (32). One possible reason could be that older adults living alone would have less social support available. Social support refers to support accessible to individuals who socially interact with other groups, larger communities, and individuals (52). Different social support conditions could remarkably affect depression prevalence, and better social support is associated with lower depression prevalence (32, 53). Older adults would experience constant stress because their defined social roles are lacking and their health is deteriorating, leading to the declining capability of individuals to deal with stressful events and the inclination to develop a negative mental state. Hence, sufficient emotional support and positive family relations are beneficial for maintaining older individuals' psychological health. Moreover, paying particular attention to older adults living alone or bereaved and encouraging them to engage in social entertainment activities, establish an efficient social supporting system, and obtain social roles are imperative.

In addition, the current study identified that satisfaction with personal life was a potential protective factor against depression in older adults. The result was consistent with previous evidence indicating that subjective wellbeing has a reciprocal relationship with physical health. Subjective wellbeing can be divided into the following aspects: economic wellbeing (the sense of the meaning of life and purpose), hedonic wellbeing (the feelings of pain, stress, anger, sadness, and happiness), and evaluative wellbeing (also known as life satisfaction) (40). Satisfaction with personal life in this study corresponds to evaluative wellbeing in subjective wellbeing. The current study suggested the negative influence of satisfaction in personal life on depression. According to Steptoe et al. (40), subjective wellbeing and physical health have a bidirectional relationship. Older adults suffering from chronic lung disease, arthritis, and coronary heart disease, reportedly have an impairment in subjective wellbeing and intensified depressive mood. Moreover, wellbeing may be protective for health maintenance (40). According to the result, health and economic policies should include the subjective wellbeing of older adults as an important objective.

Nevertheless, this study has several limitations. First, all the research participants were recruited from urban community residents in Shenzhen. Future studies should pay attention to these characteristics when generalizing the current findings. Second, this study used a cross-sectional research method that could only explore the correlation of exploratory variables. However, the pathway of causality still needs to be confirmed by longitudinal cohort studies. Third, this study adopted GDS- a screening tool instead of a clinical diagnosis for depression. As a result, the research findings only reflect the relationship between depressive symptoms and physical frailty instead of the depression diagnosed through structured clinical evaluation. Future studies should continue to collect longitudinal data to investigate the causal relationship between physical frailty and depression. Although the Katz-ADL is widely used for physical function measurement, it may not be sufficiently comprehensive. Future studies could involve more complex measures, such as physical performance and muscle strength tests to measure physical function.

## 5. Conclusion

In conclusion, this study investigated the common prevalence of depressive symptoms among community-dwelling older adults in Shenzhen. Our study indicates the critical role of physical frailty and function on depressive symptoms. Besides interventions on improving frailty and ADL, special attention should also be paid to psychological health. Psychosocial support should be provided to older adults who are living alone and with poorer wellbeing. Future research needs to explore the potential mechanisms concerning the pathophysiology of depression related to physical function and frailty.

## Data availability statement

The raw data supporting the conclusions of this article will be made available by the authors, without undue reservation.

## Ethics statement

The studies involving human participants were reviewed and approved by the Ethics Committee of the Medical College of Shenzhen University. The patients/participants provided their written informed consent to participate in this study.

## Author contributions

QW: study concept and design, data collection and analysis, quality control, interpretation of data, and preparation of the manuscript. DS: study design, quality control, interpretation of data, and preparation of the manuscript. QL: data collection and analysis and quality control. AT: data collection and analysis, interpretation of data, and preparation of the manuscript. YZ and XL: study design, data collection and analysis, and quality control. XQ: study design, data collection, quality control, and preparation of manuscript. All authors contributed to the article and approved the submitted version.

## References

[1] World Health Organization. Ageing and Health. (2022). Available online at: https://www.who.int/news-room/fact-sheets/detail/ageing-and-health (accessed December 22, 2022).

[2] World Health Organization. Mental Health of Older Adults. (2018). Available online at: https://www.who.int/news-room/fact-sheets/detail/mental-health-of-older-adults (accessed December 22, 2022).

[3] Malhi GS Mann JJ. Depression. Lancet. (2018) 392:2299–312. 10.1016/S0140-6736(18)31948-2 30396512

[4] Van Damme A Declercq T Lemey L Tandt H Petrovic M. Late-life depression: issues for the general practitioner. Int J Gen Med. (2018) 11:113–20. 10.2147/IJGM.S154876 29636629 PMC5880181

[5] Morley JE Vellas B van Kan GA Anker SD Bauer JM Bernabei R. Frailty consensus: a call to action. J Am Geriatr Soc. (2013) 14:392–7. 10.1016/j.jamda.2013.03.022 23764209 PMC4084863

[6] Soysal P Veronese N Thompson T Kahl KG Fernandes BS Prina AM. Relationship between depression and frailty in older adults: a systematic review and meta-analysis. Ageing Res Rev. (2017) 36:78–87. 10.1016/j.arr.2017.03.005 28366616

[7] Mezuk B Edwards L Lohman M Choi M Lapane K. Depression and frailty in later life: a synthetic review. Int J Geriatr Psychiatry. (2012) 27:879–92. 10.1002/gps.2807 21984056 PMC3276735

[8] Buigues C Padilla-Sanchez C Garrido JF Navarro-Martinez R Ruiz-Ros V Cauli O. The relationship between depression and frailty syndrome: a systematic review. Aging Ment Health. (2014) 19:762–72. 10.1080/13607863.2014.967174 25319638

[9] Vaughan L Corbin AL Goveas JS. Depression and frailty in later life: a systematic review. Clin Interv Aging. (2015) 10:1947–58. 10.2147/CIA.S69632 26719681 PMC4687619

[10] Coelho-Junior HJ Uchida MC Gonçalves IO Calvani R Rodrigues B Picca A. Age- and gender-related changes in physical function in community-dwelling Brazilian adults aged 50 to 102 years. J Geriatr Phys Ther. (2021) 44:E123–31. 10.1519/JPT.0000000000000246 31693536

[11] Dugan SA Gabriel KP Lange-Maia BS Karvonen-Gutierrez C. Physical activity and physical function: moving and aging. Obstet Gynecol Clin North Am. (2018) 45:723–36. 10.1016/j.ogc.2018.07.009 30401553 PMC6226270

[12] Patrizio E Calvani R Marzetti E Cesari M. Physical functional assessment in older adults. J Frailty Aging. (2021) 10:141–9. 10.14283/jfa.2020.61 33575703

[13] Kojima G. Frailty as a predictor of hospitalisation among community-dwelling older adults: a systematic review and meta-analysis. J Epidemiol Community Health. (2016) 70:722–9. 10.1136/jech-2015-206978 26933121

[14] Kojima G. Frailty defined by frail scale as a predictor of mortality: a systematic review and meta-analysis. J Am Med Dir Assoc. (2018) 19:480–3. 10.1016/j.jamda.2018.04.006 29793675

[15] Kojima G. Quick and simple frail scale predicts incident activities of daily living (ADL) and instrumental ADL (IADL) disabilities: a systematic review and meta-analysis. J Am MedDir Assoc. (2018) 19:1063–8. 10.1016/j.jamda.2018.07.019 30206033

[16] Kojima G Iliffffe S Jivraj S Walters K. Association between frailty and quality of life among community-dwelling older adults: a systematic review and meta-analysis. J Epidemiol Community Health. (2016) 70:716–21. 10.1136/jech-2015-206717 26783304

[17] Kojima G Liljas A Iliffffe S Walters K. Prevalence of frailty in mild to moderate Alzheimer's disease: a systematic review and meta-analysis. Curr Alzheimer Res. (2017) 14:1256–63. 10.2174/1567205014666170417104236 28413984

[18] Angulo J El Assar M Álvarez-Bustos A Rodríguez-Mañas L. Physical activity and exercise: Strategies to manage frailty. Redox Biol. (2020) 35:101513. 10.1016/j.redox.2020.101513 32234291 PMC7284931

[19] Choi KW Stein MB Nishimi KM Ge T Coleman JRI Chen CY. An exposure-wide and mendelian randomization approach to identifying modifiable factors for the prevention of depression. Am J Psychiatr. (2020) 177:944–54. 10.1176/appi.ajp.2020.19111158 32791893 PMC9361193

[20] Cohen J. Statistical Power Analysis for the Behavioral Sciences. Hillsdale, NJ, USA: L. Erlbaum Associates. (1988). p. 567.

[21] Hoyl MT Alessi CA Harker JO Josephson KR Pietruszka FM Koelfgen M. (1999). Development and testing of a five-item version of the geriatric depression scale. J Am Geriatr Soc. (1999) 47:873 -8. 10.1111/j.1532-5415.1999.tb03848.x 10404935

[22] Weeks SK McGann PE Michaels TK Penninx BWJH. Comparing various short-form geriatric depression scales leads to the GDS-5/15. J Nurs Scholarsh. (2003) 35:133–7. 10.1111/j.1547-5069.2003.00133.x 12854293

[23] Liu XY. The Relationship Between Sleep and Physical Frailty Among Chinese Community Dwelling Older Adults: Depression as a Mediator and Its Interaction With Sleep. Shandong University. (2020). (in Chinese). 10.27272/d.cnki.gshdu.2020.00493033836251

[24] Lee YH Lu PY Chang YC Shelley M Lee YT Liu CT. Associations of alcohol consumption status with activities of daily living among older adults in China. J Ethn Subst Abuse. (2019) 20:428–43. 10.1080/15332640.2019.1664961 31530097

[25] Morley JE Malmstrom TK Miller DK. A simple frailty questionnaire(FRAIL) predicts outcomes in middle aged African Americans. J Nutr Health Aging. (2012) 16:601e608. 10.1007/s12603-012-0084-2 22836700 PMC4515112

[26] Dong L Qiao X Tian X Liu N Jin Y Si H. Cross-Cultural adaptation and validation of the FRAIL scale in chinese community-dwelling older adults. J Am Med Dir Assoc. (2017) 19:12–7. 10.1016/j.jamda.2017.06.011 28757330

[27] Katz S Downs TD Cash HR Grotz RC. Progress in development of the index of ADL. Gerontologist. (1970) 10:20–30. 10.1093/geront/10.1_Part_1.20 5420677

[28] Hartigan I. A comparative review of the Katz ADL and the Barthel Index in assessing the activities of daily living of older adults. Int J Older adults Nurs. (2007) 2:204–12. 10.1111/j.1748-3743.2007.00074.x 20925877

[29] Lee YH Kong DX Lee YTH Lin CH Liu CT Chang YC. Functional disabilities and changes in sleep quality and duration among older adults: results from a longitudinal study in China, 2005–2014. Euro Geriatr Med. (2022) 13:967–75. 10.1007/s41999-022-00619-3 35191012

[30] Li XS Wang JJ Dong SH Fu JP Liu JP. The influence of disabilities in activities of daily living on successful aging: The role of well-being and residence location. Front Public Health. (2020) 7:417. 10.3389/fpubh.2019.00417 32047732 PMC6997131

[31] Tabachnick BG Fidell LS. Using Multivariate Statistics. 6th ed. Pearson Education Inc.: Boston, MA, USA. (2013).

[32] Liu A Peng Y Zhu W Zhang Y Ge S Zhou Y. Analysis of factors associated with depression in community-dwelling older adults in Wuhan, China. Front Aging Neurosci. (2021) 13:743193. 10.3389/fnagi.2021.743193 34867276 PMC8636125

[33] Zhao WY Zhang Y Liu XL Yue JR Hou LS Xia X. Comorbid depressive and anxiety symptoms and frailty among older adults: fifindings from the West China health and aging trend study. J Affffect Disord. (2020) 277:970–6. 10.1016/j.jad.2020.08.070 33065841

[34] Shimada H Lee S Doi T Bae S Tsutsumimoto K Arai H. Prevalence of psychological frailty in Japan: NCGG-SGS as a Japanese national cohort study. J Clin Med. (2019) 8:e8101554. 10.3390/jcm810 31569684 PMC6832757

[35] Glaesmer H Riedel-Heller S Braehler E Spangenberg L Luppa M. Age- and gender-specifific prevalence and risk factors for depressive symptoms in the elderly: a population-based study. Int Psychogeriatr. (2011) 23:1294–300. 10.1017/S1041610211000780 21729425

[36] Jorm AF. Does old age reduce the risk of anxiety and depression? A review of epidemiological studies across the adult life span. Psychol Med. (2000) 30:11–22. 10.1017/S0033291799001452 10722172

[37] Kang HJ Bae KY Kim SW Shin HY Shin IS Yoon JS. Impact of anxiety and depression on physical health condition and disability in an elderly Korean population. Psychiatry Investig. (2017) 14:240–8. 10.4306/pi.2017.14.3.240 28539942 PMC5440426

[38] Niles AN O'Donovan A. Comparing anxiety and depression to obesity and smoking as predictors of major medical illnesses and somatic symptoms. Health Psychol. (2019) 38:172–81. 10.1037/hea0000707 30556708 PMC6592048

[39] Chowdhury R Peel NM Krosch M Hubbard RE. Frailty and chronic kidney disease: a systematic review. Arch Gerontol Geriatr. (2017) 68:135–142. 10.1016/j.archger.2016.10.007 27810661

[40] Steptoe A Deaton A Stone AA. Subjective wellbeing, health, and ageing. Lancet. (2015) 385:640–8. 10.1016/S0140-6736(13)61489-0 25468152 PMC4339610

[41] Zis P Daskalaki A Bountouni I Sykioti P Varrassi G Paladini A. Depression and chronic pain in the elderly: links and management challenges. Clin Interv Aging. (2017) 12:709–20. 10.2147/CIA.S113576 28461745 PMC5407450

[42] Ferrucci L Fabbri E. Inflammageing: chronic inflammation in ageing, cardiovascular disease, and frailty. Nat Rev Cardiol. (2018) 15:505–22. 10.1038/s41569-018-0064-2 30065258 PMC6146930

[43] Woods NF LaCroix AZ Gray SL Aragaki A Cochrane BB Brunner RL. Frailty: emergence and consequences in women aged 65 and older in the women's health initiative observational study. J Am Geriatr Soc. (2005) 53:1321–30. 10.1111/j.1532-5415.2005.53405.x 16078957

[44] Lohman M Dumenci L Mezuk B. Depression and frailty in late life: evidence for a common vulnerability. J Gerontol B Psychol. (2015) 71:630–40. 10.1093/geronb/gbu180 25617399 PMC4903031

[45] Dowlati Y Herrmann N Swardfager W Liu H Sham L Reim EK. A Meta-analysis of cytokines in major depression. Biol Psychiatry. (2010) 67:446–57. 10.1016/j.biopsych.2009.09.033 20015486

[46] Duivis HE Vogelzangs N Kupper N de Jonge P Penninx BWJH. Differential association of somatic and cognitive symptoms of depression and anxiety with inflammation: findings from the Netherlands Study of Depression and Anxiety (NESDA). Psychoneuroendocrinology. (2013) 38:1573–85. 10.1016/j.psyneuen.2013.01.002 23399050

[47] Howren MB Lamkin DM Suls J. Associations of depression with c-reactive protein, IL-1, and IL-6: a meta-analysis. Psychosom Med. (2009) 71:171–86. 10.1097/PSY.0b013e3181907c1b 19188531

[48] O'Donovan A Hughes BM Slavich GM Lynch L Cronin MT O'Farrelly C. Clinical anxiety, cortisol and interleukin-6: evidence for specificity in emotion–biology relationships. Brain Behav. Immun. (2010) 24:1074–7. 10.1016/j.bbi.2010.03.003 20227485 PMC4361085

[49] Schaap LA Pluijm SMF Deeg DJH Harris TB Kritchevsky SB Newman AB. Higher inflammatory marker levels in older persons: associations with 5-year change in muscle mass and muscle strength. J Gerontol. (2009) 64:1183–9. 10.1093/gerona/glp097 19622801 PMC2759573

[50] Brown PJ Rutherford BR Yaffe K Tandler JM Ray JL Pott E. The depressed frail phenotype: the clinical manifestation of increased biological aging. Am J Geriatr Psychiatry. (2016) 24:1084–94. 10.1016/j.jagp.2016.06.005 27618646 PMC5069140

[51] Hao QK Li J Dong BR Li XY. Chinese expert consensus on frailty assessment and intervention of elderly patients. Chin J Geriatr. (in Chinese) (2017) 36:251–6. 10.3760/cma.j.issn.0254-9026.2017.03.00728238055

[52] Lin N Simeone RS Ensel WM Kuo W. Social support, stressful life events, and illness: a model and an empirical test. J Health Soc Behav. (1979) 20:108–19. 10.2307/2136433 479524

[53] Simkhada R Wasti SP Gc VS Lee A. Prevalence of depressive symptoms and its associated factors older adults: a cross-sectional study in Kathmandu. Nepal Aging Ment Health. (2018) 22:802–7. 10.1080/13607863.2017.1310803 28393547

